# Diet, Weight Status, and Pregnancy Outcomes Among Native and Migrant Women in Jersey, Channel Islands

**DOI:** 10.3390/nu17233742

**Published:** 2025-11-28

**Authors:** Urszula Śliwka, Anna Danielewicz, Neil MacLachlan, Julie Lemprière, Katarzyna E. Przybyłowicz, Justyna Borawska-Dziadkiewicz

**Affiliations:** 1 Maternity, Jersey General Hospital, Gloucester Street, St Helier JE1 3QS, UK; urszula2411@gmail.com; 2 Department of Human Nutrition, University of Warmia and Mazury in Olsztyn, ul. Sloneczna 24F, 10-718 Olsztyn, Poland; anna.danielewicz@uwm.edu.pl (A.D.); katarzyna.przybylowicz@uwm.edu.pl (K.E.P.); 3 Department of Obstetrics and Gynecology, Jersey General Hospital, Gloucester Street, St Helier JE1 3QS, UK; n.maclachlan@me.com; 4 Health and Community Services, Faculty of Health Education, Peter Crill House, Gloucester Street, St Helier JE1 3QS, UK; j.lempriere@health.gov.je; 5 Department of Food Biochemistry, University of Warmia and Mazury in Olsztyn, pl. Cieszynski 1, 10-726 Olsztyn, Poland

**Keywords:** pregnancy, nutrition, BMI, breastfeeding, ethnicity, public health

## Abstract

**Background/Objectives**: The nutrition and lifestyle choices made during pregnancy significantly impact the long-term health of both mother and child. This observational study, conducted at the Jersey Maternity Unit, investigated how maternal diet and weight influence delivery outcomes and newborn feeding methods among 81 pregnant women. The aim of the study was to assess whether maternal diet, weight changes, and infant feeding practices vary according to ethnicity and duration of residence in Jersey, in order to inform culturally sensitive health improvement strategies. **Methods**: The FFQ was used to characterise the diet before and during pregnancy. Diet quality was assessed by the 9-item aMED score. Data on anthropometric measurements (including gestational weight gain, GWG), socioeconomic characteristics, and infant feeding methods (including breastfeeding rates) were collected. **Results**: The statistical analyses evaluate differences and distribution of data in the groups of women based on the ethnicity and period the mothers had lived on the island. For all the women, there were significant changes in the weight gained at week 28 compared to weeks 8 and 12 (+8.0 (4.0; 12.1) kg, *p* ≤ 0.001), as well as the period before pregnancy until the 34th week of gestation (+11.9 (8.0; 12.1) kg, *p* ≤ 0.001). The intake of low-fat dairy, whole grains, and fruit changed through the gestation period and differed between pregnant women with different periods of island residence. There were no changes in the aMED score and adherence. **Conclusions**: Maternal weight gain followed a consistent pattern during pregnancy, whereas dietary behaviours—particularly the consumption of key food groups—varied by cultural background and length of residence. These findings emphasise the need for culturally sensitive antenatal care and tailored nutrition strategies to improve maternal outcomes, support breastfeeding, and reduce long-term health risks for mothers and children in Jersey’s multicultural population.

## 1. Introduction

Maternal nutritional status, body composition, and vitamin deficiencies are commonly studied, building the evidence for existing and future prevention programmes and public health strategies to reduce health inequalities among women and to optimise the short and long-term health outcomes for both mother and child. Nutritional and lifestyle factors in pre-pregnancy and pregnancy, maternal body mass index (BMI), maternal gestational weight gain (GWG), prenatal maternal tobacco smoking, high infant birth weight, and Intrauterine Growth Restriction (IUGR), as well as high infant weight gain, have been implicated in shaping childbirth and child health from different perspectives [1,2,3,4,5].

Public health policies addressing the increasing cost of living and nutrition-related health challenges at a population level should consider the ongoing impacts of global modernisation and migration. These factors have significant consequences for maternal and child health outcomes, particularly among groups experiencing structural disadvantages related to education and health literacy, as well as migrant populations. Large-scale migration has emerged as a social determinant of health, associated with higher incidences of adverse outcomes, especially among lower socioeconomic status (SES) [6,7,8,9,10]. This underscores health inequalities, barriers to healthcare access, and perceptions of health and well-being, as well as diminished trust in governing institutions and heightened stress among populations with lower educational attainment, limited acculturation, or minority ethnic status during periods of intensive immigration [11,12,13,14].

An obesogenic environment, as described by the World Health Organisation (WHO), is characterised by low physical activity levels and a food system dominated by highly processed, energy-dense foods high in fat, sugar, and salt, which are readily available and convenient. This makes prevention efforts even more challenging [15,16,17]. Major UK and international reports, including the Report on Inequalities in Health, UNICEF guidance, and the Early Years Healthy Development Review, have highlighted early-life nutrition and weight as key targets for reducing future nutrition-related morbidities [15,16,17]. According to WHO data, overweight and obesity affect nearly 60% of adults and one-third of children in Europe [18], and children with at least one parent or caregiver who has excess body weight have a higher risk of developing obesity. Currently, in England, 64.0% of adults are overweight or living with obesity, showing an upward trend when compared to 2015/2016 (61.2%) [19]. Creating an obesogenic environment in homes can encourage children and adolescents to imitate these patterns, develop overweight and obesity at an early age, and then, as stated by Simmonds et al. [20], increase their risk of weight-related health challenges as adults nearly fivefold.

Approximately one-fifth of women achieve adequate GWG, while nearly half experience excessive GWG, which may be carried into subsequent pregnancies [21]. This increases the risk of poor maternal health outcomes, such as higher rates of instrumental delivery, caesarean section, gestational hypertension, preeclampsia, preterm delivery, gestational diabetes, and adverse neonatal outcomes, including IUGR and an increased risk of childhood obesity [22,23,24,25,26,27,28,29,30].

Recent evidence indicates that dietary patterns high in energy-dense, ultra-processed foods, red meat, and sugar-sweetened beverages and low in fruits, vegetables, legumes, nuts, and dairy are associated with an increased risk of overweight and obesity in adults [31]. In pregnancy, greater adherence to Western-type or mixed dietary patterns rich in ultra-processed, high-fat and high-sugar foods has been associated with excessive GWG, whereas prudent, traditional or Mediterranean-type patterns with higher intakes of fruits, vegetables, whole grains, legumes, nuts and dairy are related to more adequate GWG and better maternal–child outcomes [32].

In this context, breastfeeding is at the forefront of public health agendas because of its substantial benefits for both infants and mothers. In infants, it helps prevent early-life obesity, whereas in women it supports postpartum weight management and is associated with a reduced risk of chronic conditions, including hypertension, diabetes mellitus, hyperlipidemia, cardiovascular disease, and breast cancer [33,34,35,36,37,38,39,40].

Jersey has a growing, ethnically diverse population, with native-born and British women typically attaining higher levels of education and household income compared to many recent European migrants, particularly during their initial years of residency, which limits access to stable income and housing support. Migration status and ethnicity are known determinants of access to and utilisation of healthcare services and have been linked to differences in maternal outcomes and newborn care practices, including breastfeeding [41,42]. These socioeconomic and healthcare differences suggest that native and migrant women in Jersey may experience pregnancy and early infant feeding within different contexts, which justifies our focus on ethnicity and duration of residence.

Research indicates that longer residence among immigrants is linked to an increased risk of both planned and unplanned caesarean sections [43,44]. Similar patterns have been reported in the UK and Norway [45,46], suggesting the need for individualised approaches to improve delivery outcomes. In Jersey, the caesarean section rate was 42% from 2021 to 2023, compared to 35% in England during 2021–2022. Between 2017 and 2020, Jersey’s rate was 34%, while England’s was 27% [47]. Recent regulations allowing women in Jersey and the UK to request elective C-sections may further increase these rates. Moreover, the proportion of caesarean sections has been shown to rise with maternal age. Therefore, delivery outcomes constitute a key component of maternal health that should be considered when evaluating population-level differences and trends.

In the 2008 Annual Report [48], the Medical Officer of Health in Jersey highlighted that reducing obesity among pregnant women is crucial due to the observed high percentage of women who had excess weight at the first antenatal appointment (32% overweight and 10% obese). The Public Health Jersey Obesity, Diet and Physical Activity Report from 2023 [49] presented an adult obesity rate of 50% in 2022 and 54% in 2023, where overweight or obese females were 48% and males 59%. Against this background, an observational study was conducted at the Jersey Maternity Unit, involving 81 pregnant women, to investigate whether maternal diet and weight influence delivery outcomes and newborn feeding methods. Particular attention was given to the multicultural diversity of the community and the duration of residence of mothers on the island of Jersey. To the authors’ knowledge, this is the first study of its kind to focus on the health and pregnancy outcomes of women of childbearing age in Jersey. Our study fills an important local data gap by providing the first systematically collected information on how maternal diet, gestational weight gain, and early infant feeding practices vary between native and migrant women.

## 2. Materials and Methods

### 2.1. Study Setting, Design, and Ethical Considerations

**Figure 1:**
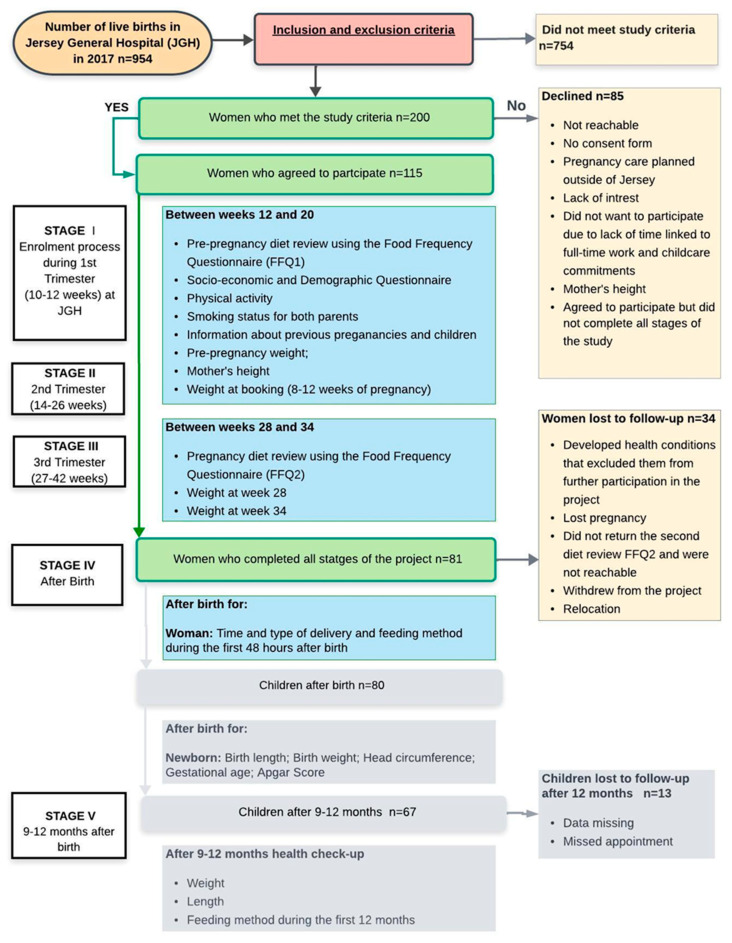
Flow chart of the study and sample collection.

The study population consisted of pregnant women who attended the Maternity Unit at Jersey General Hospital (JGH) in Jersey, the Channel Islands. The women were from various ethnic backgrounds: native-born Jersey, British, Polish, and Portuguese/Madeiran nationals. This prospective observational study was conducted over three years, between September 2016 and September 2019, accordingly to the study timeline (Figure 1). Women enrolled in this study were identified after their first appointment between the 10th and 12th weeks of pregnancy. The inclusion criteria of the study were an uncomplicated (healthy) and singleton pregnancy without gynaecological diseases; gestation before the 20th week; and ethnicity of Jersey, British, Irish, Polish, or Portuguese. Women who self-identified as Jersey or British were combined into a single “native/UK” group, reflecting the two largest ethnic categories in the source population. Other European nationalities were classified as “European migrants”. Because the individual migrant subgroups were numerically small, analyses by specific nationality were not feasible. Among European migrants, duration of residence in Jersey was categorised as <10 years and ≥10 years to capture differences in acculturation and exposure to the local health and food environment. The exclusion criteria of the study were any pathology known to alter food intake during pregnancy such as diabetes, heart disease, kidney disease, hypertension, or depression; a specific clinical issue (mental health, HIV infection, or Hepatitis B); an impairment that would limit communication during an interview, limit the patient’s legal actions, or limit their ability to provide full consent; unwillingness to participate in all stages of the research; unwillingness to have anthropometric measurements taken and used as data for the study. Therefore, women who had high-risk pregnancies were automatically excluded, which is particularly important to mention because Jersey has a population slightly greater than 103,000 [50] with only 954 live births in 2017 (in the year of recruitment) and a birth rate continuing to decline [47].

Data from the socioeconomic and demographic questionnaire (SDQ) and Food Frequency Questionnaires (FFQ) were the primary data for this study. The weight and height before pregnancy, weight during pregnancy, early pregnancy BMI trends for a wider population, the type and time of delivery, as well as a newborn’s feeding method during the first 48 h after birth, were used as secondary data. This study also involved data collection related to children’s health outcomes (Figure 1) in two stages: after birth and 12 months after birth, which will be published in the near future.

The study was reviewed and approved by the Health and Social Services Jersey Ethics Committee and, by Resolution, the Bioethics Committee of the Faculty of Medical Sciences of the UWM in Olsztyn, Poland (no. 9/2015). The processing, sharing, and publication of the results of the research were planned and conducted following the Data Protection (Jersey) Law. Participation in the study was entirely voluntary. The participants were asked to sign an informed consent form. All the information concerning the research was available in various languages: English, Portuguese, and Polish.

### 2.2. The Socioeconomic and Demographic Characteristics

The SDQ was developed as a self-reported tool to collect data about participants’ age, education level, employment status, nationality, household income, level of physical activity, as well as information related to smoking tobacco and the amount of time spent on the island for both parents. The period the mothers had lived on the island was used to split the groups of native and the UK (NU), women who had lived on the island less than 10 years (Europe < 10 y on the island or short-TLE), and women who had lived on the island more than 10 years (Europe ≥ 10 y on the island or long-TLE).

### 2.3. Anthropometric Measurements

Pre-pregnancy self-reported weight (kg) and height measurements (m) were collected from SDQ and were used to calculate the pre-pregnancy BMI and classified according to WHO recommendations [51]. The weight and height measurements for the studied cohort were obtained at each pregnancy trimester by hospital health professionals or midwives as part of the Jersey antenatal care and routine appointments following the standard techniques of the NICE Clinical Guideline for uncomplicated pregnancy [52] and the NICE Quality Standard [53]. Gestational weight gain (GWG) was calculated as the difference between the pre-pregnancy weight and the weight during the second and third trimesters. GWG was classified according to the guidelines of the US Institute of Medicine [54]. For anthropometric variables, routinely collected measurements from medical records were analysed using available-case data for each time point, without imputation of missing values.

Additionally, early pregnancy BMIs of all women who attended JGH at 8–12 weeks of gestation between 2008 and 2019 were obtained as a background for this study and to investigate the local trends. Data were provided by the Jersey Health Statistics Team based on data captured in the health system.

### 2.4. Diet Review

#### Dietary Intake Before and During Pregnancy

The EPIC-NorfolkFFQ v.6 (EPIC-Norfolk Study, 1988) was used to obtain a retrospective diet review before pregnancy (FFQ1), and during pregnancy (FFQ2) directly from participants, and both were self-reported tools. The semi-quantitative EPIC-Norfolk FFQ includes 130 food items. The food items are either individual foods, combinations of particular foods, or food types. Each item has a given portion size: a medium serving, standard unit, or household measure. The study participants were asked to select an appropriate frequency of consumption for each item from nine frequencies, from ‘Never or less than once per month’ (1) to ‘6+ per day’ (9). Part 2, ‘Back of the FFQ’, contains further questions that request more detailed information about food, e.g., food preparation and cooking methods. The FFQ1 was used to assess the diet 3 to 6 months before pregnancy and was provided for self-administered answering at the beginning of the pregnancy. The FFQ2 was dedicated to diet during pregnancy and was sent by post to each woman’s home address. The participants were asked to fill in this second diet review between week 28 and week 34 of pregnancy. Once the FFQs had been returned to the author, any missing or confusing answers were addressed and clarified directly with the participants.

### 2.5. Dietary Pattern

Maternal dietary habits were described by the degree of adherence to the alternate Mediterranean diet (aMED) score constructed by Fung et al. [55]. The aMED score is composed of eight food items (vegetables [excluding potatoes], fruits, nuts, legumes, fish, whole grains, red and processed meat, and alcohol) and the MUFA:SFA ratio. One point was awarded when the daily consumption of vegetables, fruits, nuts, legumes, fish, whole grains, and the MUFA:SFA ratio was greater than or equal to the median, and when the daily consumption of red and processed meat was less than the median, and alcohol intake ranged from 5 to 15 g per day. Adherence to the aMED score was based on the overall sum of the scored components and ranged from 0 to 9. The aMED provides an overall pattern-based indicator of diet quality rather than a comprehensive assessment of all nutrient requirements in pregnancy.

### 2.6. Other Pregnancy Outcomes: Type and Time of Delivery and the Newborn’s Feeding Methods

The type of delivery was classified as normal delivery, assisted delivery, elective caesarean section, or emergency caesarean section. The time of delivery was classified according to hospital records and the NICE guidelines [52] as preterm [37 weeks or less], term [between weeks 38 and 41], or post-term [after week 42] and recorded by hospital health professionals or midwives.

Data recording the newborn’s feeding methods during the first 48 h were classified according to the hospital records: bottle, breast only, and breast with complement.

### 2.7. Statistical Analyses

The normality of the variables was examined using the Shapiro–Wilk test. The data are presented as the median and interquartile range (IQR) for continuous variables or number and % for qualitative data. A one-way variance analysis (ANOVA) with Tukey’s post hoc test was used for the continuous variables with a normal distribution. For the continuous variables with a non-normal distribution, the Kruskal–Wallis test was applied. The distribution of qualitative variables was analysed by the Chi-square test or Fisher’s exact test. The change in food intake, dietary pattern, and weight gain before and during pregnancy was analysed with the Wilcoxon matched-pairs test. Changes in maternal weight were measured or modelled at four time points: before pregnancy, at booking (8–12 weeks of pregnancy), at 28 weeks, and at 34 weeks. The level of statistical significance was set to *p*  <  0.05 for all tests. Given the modest sample size and three-group comparisons, multivariable models with extensive adjustment were not fitted, and the results are presented primarily as unadjusted group comparisons, which should be regarded as exploratory. Statistical analyses were performed using TIBCO^®^ Statistica™ ver. 13.3 (TIBCO Software Inc., Tulsa, OK, USA) and R software (version 3.5.2, R Foundation for Statistical Computing, Vienna, Austria).

## 3. Results

### 3.1. Socioeconomic and Demographic Characteristics of the Study Population

The detailed results from SDQ are presented in Table 1. Significant differences were observed in the socioeconomic and demographic data collected, which were related to the time women spent on the island. Of the women included in the study, 68% were NU, while short-TLE women comprised 17%, and long-TLE women 15%. The higher education level was declared by 80% of NU women and 64.3% of short-TLE, while more than half (58.3%) of long-TLE had secondary education (*p* = 0.02). Significant differences were also observed in the distribution of household income. A household income of £550 or more per week was observed in 81.5% of the NU women, 36.4% in long-TLE, and 21.4% in short-TLE (*p* < 0.001). In this group, 28.6% of women declared a weekly household income of £200–400. Also, in both groups of European immigrant mothers, more than a quarter declined to answer, while only 7.4% of the NU. A total of 3.8% of women led a sedentary lifestyle, while 36.3% exercised two to three times a week. A total of 8.6% of women smoked during pregnancy.

### 3.2. Anthropometric Characteristics and Pregnancy Outcomes

The anthropometric characteristics, including the differences in weight gain at different stages of the gestational period for native and British women and women who had lived on the island for less than or more than 10 years, are presented in Table 2. Higher BMIs were recorded among long-TLE women. On average, 9.9% of the women were underweight, while 16.0% and 6.2% of the women were overweight and obese, respectively. None of the short-TLE women were obese. However, none of the analysed parameters revealed statistically significant differences. Figure S1 shows the trends in the BMIs of women who attended JGH at 8–12 weeks of gestation between 2008 and 2019. While the proportion of pregnant women in Jersey who have a healthy weight has declined over the years, the prevalence of overweight and obese pregnant women has increased.

The weight gained among the groups of women who participated in the research varied significantly based on the length of time living on the island. For all women, there were significant changes in the weight gained at week 28 compared to weeks 8 and 12 (*p* < 0.001), as well as the period before pregnancy until the 34th week of gestation (*p* < 0.001). The same trend was observed for groups of NU women and long-TLE women. For short-TLE women, significant weight gain was only observed at the 34th week of gestation (*p* < 0.001).

No significant differences were observed in terms of type and timing of delivery and feeding methods among mothers living on the island for different lengths of time (Table S1). More than half of natives and immigrants who had lived on the island for less than 10 years had spontaneous vertex delivery, but only a quarter of immigrants who had lived on the island for more than 10 years. On average, 91.3% of women had a term delivery and 61.3% offered continuous breastfeeding for the first 48 h.

Although we collected and reported data on several potential confounders (including maternal age, parity, education, income, and smoking status), the relatively small sample size limited our ability to include them in multivariable models. As a result, residual confounding by these and other unmeasured factors cannot be excluded, and the observed associations should be interpreted with caution.

### 3.3. Dietary Intake

The dietary intake (times/day) of women before and during pregnancy is presented in Table S2. When analysing the change in the frequency of consumption of selected food groups among the total study group, there was a decrease in the consumption of vegetables (from 6.13 IQR: 4.49, 7.78 to 3.18 IQR: 4.57, 6.37, *p* ≤ 0.01) and non-alcoholic (from 3.78 IQR: 1.93, 5.50 to 3.18 IQR: 1.29, 4.58, *p* ≤ 0.05) and alcoholic (from 0.28 IQR: 0.07, 0.64 to 0.00 IQR: 0.00, 0.00, *p* ≤ 0.001) beverages during pregnancy. However, for the vegetables and non-alcoholic beverages, these differences lose their significance when analysed separately within groups based on the length of time living on the island. No alcoholic beverages were consumed during pregnancy in most of the groups except for the long-TLE women, who had an intake of 0.04 (IQR: 0.00–0.07) times/day. The intake of low-fat dairy was significantly higher before pregnancy among short-TLE women (0.89 IQR: 0.28, 1.14) compared with NU women (0.28 IQR: 0.14, 0.64). A significant, double increase in the daily consumption of whole grain products during pregnancy was observed in long-TLE women (0.61 IQR: 0.39, 1.15 to 1.22 IQR: 0.57, 2.00, *p* ≤ 0.05). Compared with the pre-pregnancy intake of potatoes, during pregnancy, it increased in NU women (from 0.57 IQR: 0.35, 0.71 to 0.64 IQR: 0.43, 0.78, *p* ≤ 0.05) and decreased in short-TLE women (from 0.68 IQR: 0.28, 1.07 to 0.50 IQR: 0.28, 0.71, *p* ≤ 0.05). The frequency of fruit intake significantly increased during pregnancy for NU women (from 2.43 IQR: 1.35, 3.36 to 2.50 IQR: 1.60, 3.54, *p* ≤ 0.05). Furthermore, short-TLE women consumed fruits more frequently than the NU women before (4.33 IQR: 2.14, 6.16 vs. 2.43 IQR: 1.35, 3.36) as well as during pregnancy (3.93 IQR: 2.56, 8.28 vs. 2.50 IQR: 1.60, 3.54).

### 3.4. The Dietary Pattern

Regarding the Mediterranean diet (aMED) score and distribution of adherence to it, no significant differences were observed for the length of time spent living on the island, as well as before or during pregnancy (Table 3). However, the aMED score slightly decreased during pregnancy in short-TLE women. No relationships were observed between the maternal pregnancy parameters and the aMED score. Neither weight nor BMI was different among the aMED score levels. The aMED score was also not associated with the type or time of delivery or feeding method after birth. Table 4 summarises these exploratory associations between aMED score levels and pregnancy characteristics, illustrating how overall adherence to a Mediterranean-type dietary pattern relates to maternal anthropometric measures and selected birth outcomes in this cohort.

## 4. Discussion

### 4.1. Demographics and Lifestyle

Out of 200 pregnant women, 115 met the enrolment criteria and were willing to participate, giving the completion rate as 70% and 41% over the one-year and three-year study period, respectively. This positive level of interest among pregnant women aligns with previous findings [56], indicating that pregnancy often motivates information-seeking behaviours related to diet and lifestyle. Such behaviours create an important window of opportunity for health improvement interventions.

According to the period of time the mothers had lived on the island, the majority of actual participants of the study were Jersey Island natives and UK migrants, which was expected according to data from the Jersey Census 2021 [50]. Generally, a high level of literacy was observed among participants, with only 3.7% lacking a formal education. Nevertheless, the level of education and the household income among Jersey Island natives and UK migrants were significantly higher than those of the other European immigrants on the island. The highest proportion of participants (87.7%) had regular employment, which is also in line with the last local census, presenting a high rate (79%) of working females aged 16–59 years. This percentage was even higher (88%) in the Jersey Opinions and Lifestyle Survey [57] conducted in 2023. In the present study, 36.3% of women reported exercising two to three times per week. According to the Public Health Jersey Obesity, Diet and Physical Activity Report [49], around 55% of adults in Jersey meet the NHS guidelines for physical activity, with a slightly lower percentage of women than men. Given the significant decline in physical activity levels among women transitioning into motherhood, predisposing them to lifestyle-related diseases [58], as well as addressing local barriers and the rising BMI trends in early pregnancy, it is critical for adolescent girls and women of reproductive age to have access to guidance on nutrition and lifestyle to foster healthy habits. The preconception counselling on healthy lifestyle can also help reduce gestational weight gain (GWG), which is linked to adverse health outcomes [29].

Our findings on differences in the intake of low-fat dairy products, whole and refined grains, fruits, vegetables, and animal protein sources between residence-duration and ethnicity groups are consistent with previous evidence on maternal diet and pregnancy outcomes. High intakes of refined, high-glycaemic-index carbohydrates and sugar-rich foods in pregnancy have been linked to greater maternal adiposity, a higher risk of gestational diabetes, and an increased predisposition of the offspring to an obese phenotype, whereas higher consumption of whole grains and dietary fibre is considered protective [59,60]. Similarly, very high milk intake has been associated with an elevated risk of large-for-gestational-age infants, while moderate dairy consumption appears to support more favourable foetal growth patterns [61]. Low fruit and vegetable intake has also been related to an increased risk of foetal complications, including small-for-gestational-age births [62]. Taken together, these data suggest that the suboptimal fruit and vegetable consumption and the shifts towards more refined grains and lower low-fat dairy intake observed in parts of our Jersey cohort may have clinically relevant implications for gestational weight gain and foetal growth, even if not all associations reached statistical significance in this small sample.

Although cultural and communication barriers were not directly assessed in this study, the differences that were observed between native/UK women and European migrants with different durations of residence in terms of dietary behaviours and early infant feeding practices can be interpreted as being influenced by migration- and culture-related factors. When these patterns are considered together with previously reported inequalities in access to and utilisation of maternal health services among migrant and ethnic minority women, the need for culturally responsive antenatal care in Jersey is reinforced [11,12,13,14].

### 4.2. Anthropometric Characteristics and Pregnancy Outcomes

Approximately a quarter of the pregnant women were overweight or obese at their first booking appointment, especially among those living more than 10 years on the island. Furthermore, the proportion of overweight, obese, and morbidly obese women in early pregnancy showed a general upward trend from 2008 to 2019 (Figure S1). There was only a subtle increase in the proportion of underweight women. Different studies show that women who were obese before pregnancy or gained too much weight during pregnancy were found to have deteriorated health, which directly impacts the pregnancy outcomes for themselves and their children [1,63,64] hence, for the best outcomes weight management should start well before pregnancy if not in early childhood if possible, to break out the cycle of obesity [65].

The weight gain among women participating in the study varied significantly based on the duration of their living on the island. Notably, there were significant changes in weight at week 28 compared to weeks 8 and 12 (*p* ≤ 0.001), and from the pre-pregnancy period up until week 34 (*p* ≤ 0.001). It should be noted that, in line with the study protocol, women with high-risk pregnancies, potentially associated with unhealthy weight, were automatically excluded. Nevertheless, the findings are consistent with population studies that highlight racial-ethnic differences in gestational weight gain, particularly among overweight or obese women [66]. However, most of these studies were conducted in regions with distinct racial-ethnic populations, such as the US, where Black–White comparisons are common. Developing a detailed local women’s health profile will support targeted strategies to improve maternal outcomes. Both inadequate and excessive weight gain during pregnancy can negatively affect the foetal environment during critical developmental stages, potentially leading to chronic diseases in later life [67]. Several developed countries (such as the US) implement the routine measurement of a woman’s weight and BMI during pregnancy [68]. Moreover, the ACOG [69] provides national guidelines for the measurement of weight and GWG. However, the UK NICE guidelines do not support routine weight measurement of pregnant women unless clinical handling will be affected or if there is a concern regarding dietary intake [70], and Jersey follows the same approach. Given the impact of pre-pregnancy weight and GWG on pregnancy outcomes, routine weight monitoring before, during, and after pregnancy could create opportunities for timely interventions, reducing health risks and supporting long-term health for both mother and child.

In this study, most mothers chose exclusive breastfeeding within the first 48 h after giving birth, which is comparable to the 2023 Jersey breastfeeding report, which recorded an 82% rate [47]. Among NU women, formula feeding was more common during the first 48 h. Supporting breastfeeding through health policy and employment laws is crucial for promoting this practice. Investing in social support, family education, and a breastfeeding-friendly environment is also crucial for achieving more sustainable outcomes and fostering positive experiences for women and families throughout their breastfeeding journey. Factors such as young age, low maternal education, low SES, and belonging to minority ethnic groups have been linked to lower breastfeeding rates [42]. Addressing local barriers to breastfeeding—such as the impact of returning to work and the need to monitor feeding practices at 4 and 6 months postpartum—is essential for improving outcomes. Enhancing breastfeeding training for healthcare professionals, for example, the General Practitioners, as well as family-focused organisations, including local charities that support low-income families and ethnic minorities, could significantly boost breastfeeding rates among underserved and harder-to-reach groups.

Beyond these structural and social factors, breastfeeding also has important nutritional and metabolic implications. From a nutritional perspective, breastfeeding has important implications for both maternal and child health. Lactation increases energy and nutrient requirements, and the composition of human milk for several micronutrients and fatty acids is partly influenced by maternal dietary intake and nutritional status [71,72,73]. Longer breastfeeding duration has been associated with a quicker return to pre-pregnancy weight and a reduced risk of obesity and cardiometabolic disease in mothers, while also protecting offspring against later obesity and other chronic conditions [39,74]. In this context, the group differences in early infant feeding practices observed in our study reinforce the need for antenatal and postnatal nutrition counselling that integrates both pregnancy and lactation, with particular attention to supporting breastfeeding and promoting overall healthy dietary patterns in mothers.

### 4.3. Food Intake and Dietary Pattern

In-depth findings on maternal diet before and during pregnancy revealed that fat and oil intake increased during pregnancy, while the consumption of milk and dairy products, particularly low-fat options, decreased. This trend was consistent across all participant groups. Research indicates that moderate dairy intake is associated with better foetal growth compared to low or no intake [61]. Although not significant, there was a general decrease or stabilisation in the intake of animal products (meat, poultry, fish, and eggs) across the cohort. This is notable given that protein needs increase by 15–25% during the second and third trimesters. The findings highlight the need for improved protein intake among pregnant women in Jersey to meet the increased demands of pregnancy. Notably, whole grain consumption increased during pregnancy, particularly among European immigrants living in Jersey for less than 10 years, who significantly reduced their intake of refined grains. This suggests healthier pre-pregnancy habits, possibly from their countries of origin [75], which they tended to lose as their stay on the island increased. Research shows that at least 175 g of carbohydrates and 28 g of fibre are necessary for the growing foetus and pregnant woman [59]. Whole grains, with their lower glycaemic index, are preferable to refined carbohydrates, which are linked to increased adiposity, gestational diabetes, and predisposition of the offspring to the obese phenotype [60]. Participants across all groups tended to increase their fruit intake while reducing vegetable consumption during pregnancy. The NICE Maternal and Child Nutrition guidelines recommend at least five portions of fruits and vegetables daily [76]. Research has demonstrated that low consumption of fruits and vegetables during pregnancy is associated with an increased risk of foetal complications, such as small-for-gestational-age offspring [62]. Therefore, it is crucial to encourage higher vegetable intake among pregnant women. The Healthy Start Scheme in Jersey, which provides vouchers for fresh fruits and vegetables, supports this recommendation, particularly benefiting low-income pregnant women both in the UK and Jersey.

Differences in dietary patterns, as measured by aMED scores, were observed across the groups, though they were not significant. Women who had lived on the island for less than 10 years generally had better dietary scores entering pregnancy, while native women or those who had lived on the island for more than 10 years saw a decline in diet quality during pregnancy. Despite these differences, a better aMED score did not correlate with a healthier BMI. These findings imply that tailored pre-pregnancy dietary advice may be beneficial, not only for specific ethnic groups but for all women, especially that in Jersey in 2023, the recommended fruit or vegetables in the previous 24 h were eaten by slightly less than a third (32%) of adults and only a quarter (26%) of people aged 16–34 years. The similar proportions were observed in previous years (2013–2019) [57]. However, these findings are limited by the small sample size and the subjective nature of dietary information, as noted in the study limitations.

Dietary habits established in childhood are strongly shaped by the family food environment and parental role modelling, and they tend to track into adolescence and adulthood [77,78]. Women of reproductive age often continue to eat in ways that reflect what was customary in their family, and these patterns are frequently carried into pregnancy and the postpartum period and then transmitted to children through shared family meals and feeding practices [78]. Importantly, these intergenerational processes may differ between native and migrant families, as food traditions, feeding norms, and how parents adapt their diets to the host food environment vary by country of origin and cultural background. This represents an important public health concern, as pregnancy and the early postnatal period offer a critical window to support parents in establishing healthier family food routines and early feeding behaviours that can positively influence the next generation [4].

Beyond nutritional guidelines, the Mediterranean diet reflects a balanced and healthy lifestyle—encompassing physical activity, rest, traditional cooking methods, and social engagement. Early nutritional assessments in pregnancy could help identify poor dietary patterns, empower women, and address barriers to healthy eating, such as financial constraints, ethnicity, cultural background, as well as the lack of knowledge related to food shopping, planning, cooking, or understanding of food labels. These efforts could also help address locally prevalent weight-related issues among adults of childbearing age, who serve as role models for their families in adopting healthy diets and lifestyles from early childhood. Strengthening parental skills is crucial for any food, nutrition, or weight-related strategy, both to reduce high childhood BMI and to support healthy postpartum weight loss, ultimately promoting long-term health for women.

Although this study was conducted in a small island setting and the absolute prevalences of overweight, obesity, and specific pregnancy outcomes are context-specific to Jersey, several of the patterns observed are likely to be relevant to other multicultural European settings. Similar to other European countries, migrant and ethnic minority women in Jersey often occupy less advantaged socioeconomic positions, face language and communication barriers, and may be less familiar with the organisation of local health services, all of which can shape their access to care, lifestyle behaviours, and pregnancy outcomes.

### 4.4. The Study Limitations and Strengths

The reliance on self-declared ethnicity may introduce bias and this is why the SDQ for this study was designed to capture information on the duration of residency or period of living in Jersey. For instance, someone born outside Jersey but having lived on the island for many years may identify as a “Jersey native.” Conversely, individuals born in Jersey with parents from other countries may define their ethnicity by their place of birth rather than their parents’ origin. Additionally, those with dual citizenship can choose which nationality to report. Future research should include samples that are representative of the broader population, and reliable ethnicity-related information could support administrative data sources in Jersey, enhancing the interpretation of local health data.

Out of the total population of 954 pregnant women who attended the maternity services in Jersey in 2017, 200 were identified as potential participants. This relatively small pool was largely the result of strict exclusion criteria imposed by the local ethics committee, which limited inclusion to women with low-risk pregnancies and restricted access to some types of clinical data. Among these 200 eligible women, 81 completed all stages of the study (40.5%), which represents a relatively high completion rate for a long dietary study, especially in pregnancy. Nevertheless, from a statistical perspective, a final sample size of 81 women is modest and inevitably reduces power, increases the imprecision of effect estimates, and limits the ability to detect smaller differences or associations, which should be taken into account when interpreting the findings. For comparison, simple sample size calculations for the underlying population of 954 pregnant women, assuming an expected prevalence of 10% for high aMED scores and 20% for overweight/obesity, a 5% margin of error, and a 95% confidence level, would suggest required sample sizes of approximately 121 and 196 women, respectively. To strengthen causal inference in this area, larger prospective cohort studies with repeated dietary assessments, more extensive adjustment for potential confounders, and, ultimately, intervention studies targeting maternal diet and gestational weight gain would be required.

An arguable limitation is the dietary assessment with the use of the FFQ, the recall bias, and the inaccuracy in determining the actual dietary characteristics of the foods. The multicultural nature of the participants made it challenging to accurately assess traditional or culturally specific foods and preparation methods. Immigrants often adapt their traditional meals to include ingredients available in the host country, complicating the FFQ’s accuracy, as described by Satia-About [79]. Therefore, it is important to use an accurate and reliable dietary assessment tool that presents minimal room for bias. The FFQ, as a self-reported tool, was approved by the Jersey ethics committee as the nutritional assessment method to avoid any additional appointments for pregnant women during this study, which limited the depth of the research findings. Although the researcher obtained approval from the Maternity Unit that anthropometric data throughout pregnancy would be collected for all participants, some records were incomplete. Moreover, since the researcher relied on the self-recorded pre-pregnancy weight, the study findings for pre-pregnancy BMI may have limited use. A further limitation is that some anthropometric measurements were missing at specific gestational weeks, and no imputation was performed, which may have reduced statistical power and contributed to imprecision in some of the estimates. Additionally, women with high-risk pregnancies related to unhealthy weight were excluded from the study, which limits the interpretation and generalisation of the results.

Although the findings should be interpreted with caution, this study has several strengths. To the best of the author’s knowledge, this is the first study to investigate maternal lifestyle factors, anthropometric measurements, and other pregnancy outcomes in relation to ethnicity and migration status in the Jersey community. As described in the research design section, the researcher collaborated with several local services and organisations to ensure the reliability of the data obtained. This study included common risk factors and outcomes, such as maternal diet and high BMI. A significant strength of this project is the inclusion of previously unpublished data on the BMI of pregnant women in Jersey from 2008 to 2019, along with current trends related to this health issue. Understanding these relationships may help develop more effective prevention and intervention programmes tailored to nutritional and health concerns for women and children, ultimately improving the health outcomes of the multinational population.

Unlike many studies that measure participants at a single point in time, this study monitored subjects throughout the research period. This approach allowed for the collection of highly accurate data regarding exposures, confounders, and endpoints. Consequently, associations between various exposures and outcomes were established. Moreover, the study developed hypotheses regarding the underlying aetiologies, which can be tested in future ‘confirmatory’ studies.

## 5. Conclusions

In conclusion, this study provides novel evidence on maternal diet, weight status, and pregnancy outcomes in Jersey’s multicultural community. We observed significant increases in maternal weight across gestation, with notable differences in dietary intake, especially the consumption of low-fat dairy, whole grains, and fruit, depending on the length of residence on the island. Despite these variations, overall diet quality, as measured by the aMED score, remained unchanged. Importantly, in all analysed groups, dietary patterns deviated from the recommended pro-health dietary standards.

Moreover, the study revealed a considerable proportion of women with BMI outside the recommended range at the beginning of pregnancy, indicating elevated risks of overweight and obesity both before and after pregnancy. These conditions, in turn, pose significant short- and long-term health risks for both mother and child. 

These findings emphasise the need for culturally sensitive antenatal care and tailored nutrition strategies aimed at improving maternal outcomes, supporting breastfeeding, and reducing long-term health risks for mothers and their children in Jersey’s multicultural population.

## Figures and Tables

**Table 1:** Socioeconomic and demographic characteristics of the women who participated in the study.

Characteristics	All	Length of Time Living on the Island			*p*
		Native and British	European ≥ 10 y on the Island	European < 10 y on the Island	
*n*	81	55	12	14	
Age, years	33.0 (30.0, 36.0)	33.0 (29.0, 36.0)	35.0 (34.5, 37.5)	31.5 (28.0, 33.0)	0.174
Residence in Jersey, years	N/A	N/A	14.0 (12.0, 16.0)	5.5 (4.0, 7.0)	<0.001
Education level, *n* (%)					
Higher	57 (70.4)	44 (80.0)	4 (33.3)	9 (64.3)	0.022
Secondary	18 (22.2)	7 (12.7)	7 (58.3)	4 (28.6)	
Other	3 (3.7)	2 (3.6)	1 (8.3)	0 (0.0)	
No formal qualification	3 (3.7)	2 (3.6)	0 (0.0)	1 (7.1)	
Employment status, *n* (%)					
Regular	71 (87.7)	47 (85.5)	11 (91.7)	13 (92.9)	0.955
Student	1 (1.2)	1 (1.8)	0 (0.0)	0 (0.0)	
Unemployed	2 (2.5)	2 (3.6)	0 (0.0)	0 (0.0)	
Housewife	7 (8.6)	5 (9.1)	1 (8.3)	1 (7.1)	
Household income, *n* (%)					
<£200	1 (1.3)	0 (0.0)	0 (0.0)	1 (7.1)	<0.001
£200–400	7 (8.9)	2 (3.7)	1 (9.1)	4 (28.6)	
£400–550	9 (11.4)	4 (7.4)	3 (27.3)	2 (14.3)	
>£550	51 (64.7)	44 (81.5)	4 (36.4)	3 (21.4)	
Declined to answer	11 (13.9)	4 (7.4)	3 (27.3)	4 (28.6)	
Physical activity, *n* (%)					
Daily exercise	16 (20.0)	11 (20.0)	4 (8.3)	4 (30.8)	0.563
Exercise 2–3 times/week	29 (36.3)	22 (40.0)	5 (41.7)	2 (15.4)	
Exercise once/week	17 (21.3)	11 (20.0)	2 (16.7)	4 (30.8)	
Occasional	15 (17.8)	9 (16.7)	4 (33.3)	2 (15.4)	
Sedentary	3 (3.8)	2 (3.6)	0 (0.0)	1 (7.7)	
Smoking before pregnancy, *n* (%)					
Non-smoker	64 (79.0)	44 (80.0)	10 (83.3)	10 (71.4)	0.849
Ex-smoker	10 (12.4)	6 (10.9)	1 (8.3)	3 (21.4)	
Current smoker	7 (8.6)	5 (9.1)	1 (8.3)	1 (7.1)	
Smoking status at booking, *n* (%)					
Non-smoker	64 (79.0)	44 (80.0)	10 (83.3)	10 (71.4)	0.849
Ex-smoker	10 (12.4)	6 (10.9)	1 (8.3)	3 (21.4)	
Current smoker	7 (8.6)	5 (9.1)	1 (8.3)	1 (7.1)	

Data are presented as the median and interquartile range (IQR) or number (%). *p* < 0.05 represents a significant difference between the time on the island groups (Kruskal–Wallis test or ANOVA with Tukey post hoc test for continuous data and Chi-square test for categorical data).

**Table 2:** Anthropometric characteristics of pregnant women.

Characteristics	All	Length of Time Living on the Island			*p*
		Native and British	European ≥ 10 y on the Island	European < 10 y on the Island	
*n*	81	55	12	14	
Height, cm	165.0 (160.0, 169.0)	166.0 (160.5, 169.0)	165.5 (161.0, 169.0)	164.5 (159.0, 170.0)	0.960
Weight					
before pregnancy, kg	59.0 (55.2, 66.0)	59.0 (55.0, 66.0)	62.5 (57.5, 72.0)	59.0 (54.5, 67.0)	0.491
at 8–12 weeks, kg	63.0 (57.0, 70.5)	61.5 (56.0, 70.0)	60.5 (59.3, 75.3)	63.3 (57.9, 71.0)	0.787
at 28 weeks kg ^†^	69.0 (63.0, 74.5)	68.3 (54.0, 73.3)	86.5 (72.0, 91.0)	70.0 (66.0, 73.1)	0.157
at 34 weeks kg ^‡^	71.5 (65.1, 76.0)	71.5 (63.3, 75.3)	75.0 (72.0, 93.8)	69.3 (65.3, 76.9)	0.293
Weight gain ^⁑^					
at 8–12 weeks vs. before pregnancy	+2.7 (0.0; 5.3) *	+2.8 (0.2; 5.4) *	+1.7 (−3.9; 7.2)	+3.4 (−1.7; 8.6)	0.693
at 28 weeks vs. at 8–12 weeks	+8.0 (4.0; 12.1) ***	+8.4 (4.6; 12.3) ***	+8.9 (1.1; 16.6) *	+6.9 (−1.6; 15.3)	0.623
at 34 weeks vs. at 28 weeks	+1.2 (−5.8; 3.4)	+1.2 (−3.1; 5.5)	+1.7 (−7.1; 10.5)	+3.0 (−6.5; 12.6)	0.534
at 34 weeks vs. before pregnancy	+11.9 (8.0; 12.1) ***	+10.0 (6.4; 13.7) ***	+12.3 (4.5; 20.0) **	+13.3 (5.7; 21.0) ***	0.774
BMI					
before pregnancy, kg/cm^2^	22.1 (20.2, 24.6)	21.5 (20.2, 23.4)	23.8 (20.5, 26.5)	22.6 (20.0, 24.2)	0.440
at 8–12 weeks, kg	22.7 (20.6, 26.2)	22.3 (20.4, 25.1)	23.2 (21.1, 27.9)	22.8 (21.0, 26.4)	0.531
BMI before pregnancy					
Underweight	8 (9.9)	6 (10.9)	1 (8.3)	1 (7.1)	0.285
Normal	55 (67.9)	39 (70.9)	5 (41.7)	11 (78.6)	
Overweight	13 (16.0)	7 (12.7)	4 (33.3)	2 (14.3)	
Obese	5 (6.2)	3 (5.5)	2 (16.7)	0 (0.0)	

Data are presented as the median and interquartile range (IQR), the average estimated disparity ^⁑^ and 95% confidence interval (95% CI) or number (%). BMI, body mass index. ^†^ *n* = 29; ^‡^ *n* = 33. *p* < 0.05 represents a significant difference between the time on the island groups(Kruskal–Wallis test, ANOVA with Tukey post hoc test); * *p* < 0.05, ** *p* < 0.01, *** *p* < 0.001—for the differences between the time of pregnancy.

**Table 3:** Mediterranean diet score distribution for women before and during pregnancy.

aMED Score and Adherence	All	Length of Time Living on the Island			*p*
		Native and British	European ≥ 10 y on the Island	European < 10 y on the Island	
Before pregnancy					
aMED score, (-)	3.0 (2.0; 4.0)	3.0 (2.0; 4.0)	3.0 (2.0; 4.5)	3.0 (3.0; 4.0)	0.749
Low, *n* (%)	47 (58.0)	31 (56.4)	8 (66.7)	8 (57.1)	0.500
Medium, *n* (%)	28 (34.6)	18 (32.7)	4 (33.3)	6 (42.9)	
High, *n* (%)	6 (7.4)	6 (10.9)	0 (0.0)	0 (0.0)	
During pregnancy					
aMED score, (-)	3.0 (2.0; 4.0)	3.0 (2.0; 4.0)	2.0 (1.0; 3.5)	4.0 (3.0; 5.0)	0.158
Low, *n* (%)	31 (62.0)	21 (65.3)	6 (75.0)	4 (40.0)	0.216
Medium, *n* (%)	16 (32.0)	9 (28.1)	1 (12.5)	6 (60.0)	
High, *n* (%)	3 (6.0)	2 (6.25)	1 (12.5)	0 (0.0)	
*p * ^†^	0.235	0.247	0.161	0.529	

Data are presented as the median and interquartile range (IQR) or number (%). aMED, alternate Mediterranean diet score. *p* < 0.05 represents a significant difference between the time on the island groups (Kruskal–Wallis test or ANOVA with Tukey post hoc test) or ^†^ before and during pregnancy (Wilcoxon matched pairs test).

**Table 4:** Pregnancy characteristics and aMED score levels.

Characteristics	aMED Score Before Pregnancy			*p*
	Low	Medium	High	
*n*	47	28	6	
Age, years	33.0 (30.0, 36.0)	33.5 (31.0, 36.5)	31,0 (29.0, 35.0)	0.924
Weight before pregnancy, kg	58.0 (54.0, 66.0)	61.0 (57.6, 69.0)	59.5 (56.0, 64.0)	0.219
Weight at 8–12 weeks, kg	60.0 (56.0, 70.5)	64.5 (60.5, 71.5)	57.5 (54.0, 64.0)	0.149
Weight at 28 weeks [kg] ^†^	72.0 (56.0, 86.5)	67.8 (64.0, 73.3)	66.4 (61.7, 71.0)	0.765
Weight at 34 weeks [kg] ^‡^	72.0 (65.3, 78.0)	70.2 (64.5, 75.2)	68.5 (62.9, 74.0)	0.698
BMI before pregnancy [kg/m^2^]	22.5 (19.9, 25.0)	22.1 (20.5, 24.9)	21.7 (20.2, 22.9)	0.756
BMI at 8–12 weeks, kg/m^2^	22.3 (21.0, 26.2)	23.2 (20.5, 26.7)	20.7 (19.8, 22.6)	0.189
BMI at 28 weeks [kg/m^2^] ^†^	26.1 (21.9, 31.0)	23.6 (22.3, 27.2)	22.9 (20.9, 24.9)	0.704
BMI at 34 weeks [kg/m^2^] ^‡^	27.3 (24.7, 31.2)	24.2 (22.8, 27.3)	23.6 (21.3, 25.9)	0.206
Delivery type, *n* (%)				
Natural (spontaneous vertex)	27 (58.7)	14 (50.0)	3 (50.0)	0.837
Assisted delivery	9 (19.6)	7 (25.0)	1 (16.7)	
Caesarean section	10 (21.7)	6 (21.4)	2 (33.3)	
Emergency caesarean section (breech)	0 (0.0)	1 (3.6)	0 (0.0)	
Delivery time, *n* (%)				
Term	41 (89.1)	26 (92.9)	6 (100.0)	0.913
Preterm	3 (6.5)	1 (3.6)	0 (0.0)	
Post-term	2 (4.4)	1 (3.6)	0 (0.0)	
Feeding method during the first 48 h, *n* (%)				
Breast only formula (Bottle)	29 (63.0)	16 (57.1)	4 (66.7)	0.827
Formula (Bottle)	9 (19.6)	4 (14.3)	1 (16.7)	
Breast and complement	8 (17.4)	8 (28.6)	1 (16.7)	

Data are presented as the median and interquartile range (IQR) or number (%). aMED, alternate Mediterranean diet score. BMI, body mass index. *p* < 0.05 represents a significant difference between the aMED score groups (Kruskal–Wallis test or ANOVA with Tukey post hoc test); ^†^ *n* = 29; ^‡^ *n* = 33.

## Data Availability

The data presented in this study are available on request from the author and principal investigator, US. The data are not publicly available due to privacy and ethical restrictions.

## Supplementary Materials

The following supporting information can be downloaded at: https://www.mdpi.com/article/10.3390/nu17233742/s1, Figure S1: BMI classification of all women at the Maternity Unit at Jersey General Hospital in 2017; Table S1: Type and time of delivery and newborn’s feeding methods; Table S2: Frequency of the intake of various food groups in women before and during pregnancy.

## Abbreviations

The following abbreviations are used in this manuscript:

ACOG	American College of Obstetricians and Gynaecologists
aMED	alternate Mediterranean diet score
ANOVA	analysis of variance
BMI	body mass index
EPIC	European Prospective Investigation into Cancer
FFQ	Food Frequency Questionnaire
GWG	gestational weight gain
IQR	interquartile range
IUGR	intrauterine growth restriction
JGH	Jersey General Hospital
long-TLE	women who had lived on the island for more than 10 years
MECC	Making Every Contact Count
MUFA	monounsaturated fatty acids
NCDs	non-communicable diseases
NHS	National Health Service
NICE	National Institute for Health and Care Excellence
NU	native and the UK group,
SDQ	socioeconomic and demographic questionnaire
SES	socioeconomic status
SFA	saturated fatty acids
short-TLE	women who had lived on the island for less than 10 years
WHO	World Health Organization
