# Supplementary material for: Diet, Weight Status, and Pregnancy Outcomes Among Native and Migrant Women in Jersey, Channel Islands
Source: Nutrients. 2025 Nov 28;17(23):3742. doi: 10.3390/nu17233742 (PMC12694125; doi:10.3390/nu17233742)
Supplement: Supplementary file 1 [file nutrients-17-03742-s001.zip › nutrients-3942739-supplementary.pdf]

## Supplementary Materials

# Diet, Weight Status and Pregnancy Outcomes among Native and Migrant Women in Jersey, Channel Islands

**Urszula Śliwka [1,2,†], Anna Danielewicz [2], Neil MacLachlan [3], Julie Lemprière [4], Katarzyna E. Przybyłowicz [2] and Justyna Borawska-Dziadkiewicz [5,*]**

[1] Maternity, Jersey General Hospital, Gloucester Street, St Helier JE1 3QS, UK; urszula2411@gmail.com
[2] Department of Human Nutrition, University of Warmia and Mazury in Olsztyn, ul. Sloneczna 24F, 10-718 Olsztyn, Poland; anna.danielewicz@uwm.edu.pl (A.D.); katarzyna.przybylowicz@uwm.edu.pl (K.E.P.)
[3] Department of Obstetrics and Gynecology, Jersey General Hospital, Gloucester Street, St Helier JE1 3QS, UK; n.maclachlan@me.com
[4] Health and Community Services, Faculty of Health Education, Peter Crill House, Gloucester Street, St Helier JE1 3QS, UK; j.lempriere@health.gov.je
[5] Department of Food Biochemistry, University of Warmia and Mazury in Olsztyn, pl. Cieszynski 1, 10-726 Olsztyn, Poland
* Correspondence: justyna.borawska@uwm.edu.pl
† The work was completed while Urszula Śliwka was studying at Department of Human Nutrition, University of Warmia and Mazury in Olsztyn, Poland.

ORCID
Urszula Śliwka 0000-0001-5316-7742
Anna Danielewicz 0000-0002-3684-4278
Neil MacLachlan
Julie Lemprière
Katarzyna E. Przybyłowicz 0000-0003-1061-8448
Justyna Borawska-Dziadkiewicz 0000-0003-4322-9211

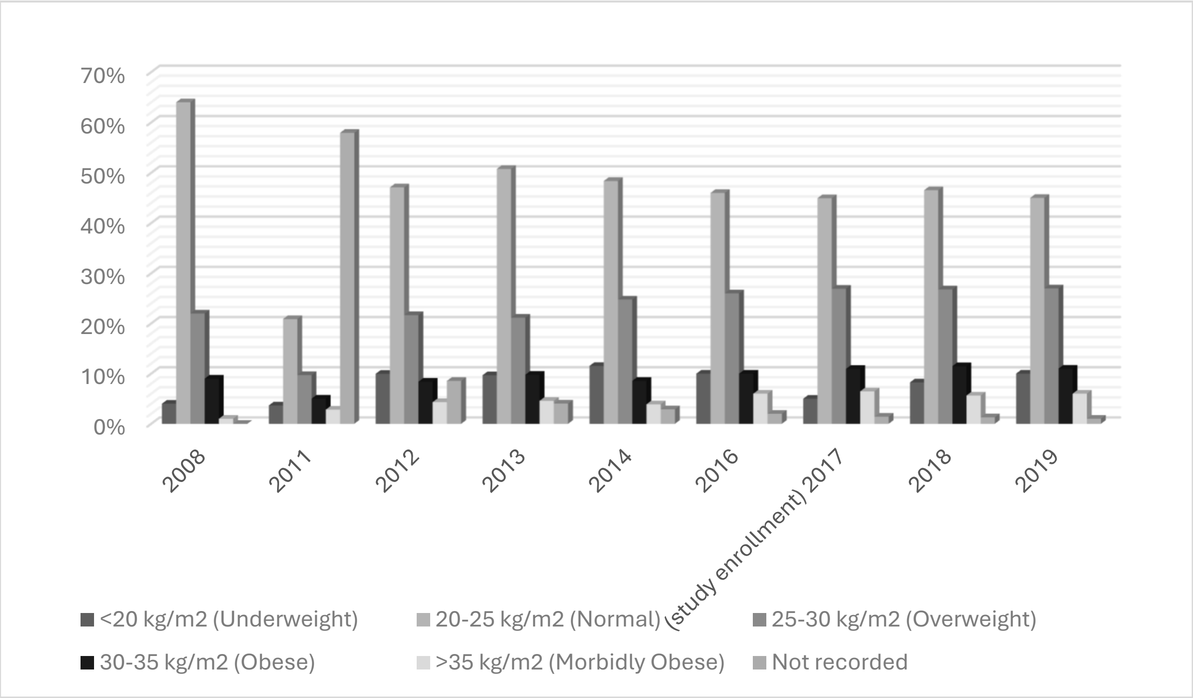


**Figure S1.** BMI classification of all women at the Maternity Unit at Jersey General Hospital in 2017 (46% of the women had a normal or healthy body weight. A significant proportion of overweight (27%), obese (11%), morbidly obese (6%) and underweight (9%). (*Source: Data for 2008 – The State of Jersey, 2008; data for 2011 to 2019 – TrakCare reports MAT00023A and MAT00017C. Accessed February 24, 2020.)*

**Table S1.** Type and time of delivery and newborn's feeding methods

| **Characteristics** | **All** | **Length of time living on the island** | | | ***p*** |
|---|---|---|---|---|---|
| | | **Native and British** | **European ≥ 10y on the island** | **European < 10y on the island** | |
| *n* | 80 | 54 | 12 | 14 | |
| Is this current pregnancy planned? [yes], n (%) | 64 (80.3) | 44 (80.0) | 11 (91.7) | 10 (71.4) | 0.691 |
| Delivery type, n (%) | | | | | |
| Natural (spontaneous vertex) | 44 (55.0) | 31 (57.4) | 3 (25.0) | 10 (71.4) | 0.152 |
| Assisted delivery | 17 (21.3) | 10 (18.5) | 6 (50.0) | 1 (7.1) | |
| Caesarean section | 18 (22.5) | 12 (22.2) | 3 (25.0) | 3 (21.4) | |
| Emergency Caesarean section (breech) | 1 (1.3) | 1 (1.9) | 0 (0.0) | 0 (0.0) | |
| Delivery time, n (%) | | | | | |
| Term | 73 (91.3) | 50 (92.6) | 11 (91.7) | 12 (85.7) | 0.262 |
| Pre-term | 4 (5.0) | 1 (1.9) | 1 (8.3) | 2 (14.3) | |
| Post-term | 3 (3.8) | 3 (5.6) | 0 (0.0) | 0 (0.0) | |
| Feeding method during the first 48-hours, n (%) | | | | | |
| Breast only | 49 (61.3) | 32 (59.3) | 7 (58.3) | 10 (71.4) | 0.669 |
| Formula (Bottle) | 14 (17.5) | 11 (20.4) | 1 (8.3) | 2 (14.3) | |
| Breast and complement | 17(21.3) | 11 (20.4) | 4 (33.3) | 2 (14.3) | |

Data are presented as the median and interquartile range (IQR) or number (%). *n=47 (native and UK = 30, Europe ≥ 10 on the island = 7, Europe < 10 on the island = 10); $p < 0.05$ represents a significant difference between the time on the island groups (chi-square test).

**Table S2.** Frequency of the intake of various food groups in women before and during pregnancy

| Intake frequencies of food groups (times/ day) | All | | Length of time living on the island | | | | | |
|---|---|---|---|---|---|---|---|---|
| | | | Native and British | | European >10y on the island | | European < 10y on the island | |
| | Before pregnancy | During pregnancy | Before pregnancy | During pregnancy | Before pregnancy | During pregnancy | Before pregnancy | During pregnancy |
| *n* | 80 | 60 | 54 | 39 | 12 | 10 | 14 | 11 |
| Meat and meat products | 1.14 (0.63, 1.57) | 0.99 (0.49, 1.21) | 1.20 (0.56, 1.57) | 0.92 (0.42, 1.13) | 0.96 (0.39, 1.61) | 0.96 (0.49, 1.57) | 1.10 (0.78, 1.71) | 1.07 (0.64, 1.34) |
| Red and processed meat | 0.70 (0.42, 1.14) | 0.57 (0.28, 0.85) | 0.71 (0.28, 1.13) | 0.49 (0.28, 0.78) | 0.57 (0.25, 1.18) | 0.67 (0.35, 1.14) | 0.49 (0.49, 1.28) | 0.64 (0.28, 1.20) |
| Poultry | 0.43 (0.14, 0.43) | 0.43 (0.14, 0.43) | 0.43 (0.14, 0.43) | 0.43 (0.14, 0.43) | 0.43 (0.14, 0.43) | 0.43 (0.00, 0.43) | 0.43 (0.14, 0.43) | 0.43 (0.14, 0.43) |
| Fish and fish products | 0.35 (0.14, 0.63) | 0.35 (0.14, 0.49) | 0.35 (0.21, 0.64) | 0.35 (0.14, 0.49) | 0.18 (0.04, 0.32) | 0.35 (0.14, 0.49) | 0.35 (0.14, 0.71) | 0.28 (0.14, 0.42) |
| Eggs and egg dishes | 0.43 (0.14, 0.50) | 0.21 (0.14, 0.43) | 0.43 (0.14, 0.50) | 0.21 (0.14, 0.43) | 0.29 (0.14, 0.47) | 0.43 (0.14, 0.43) | 0.29 (0.14, 0.43) | 0.14 (0.14, 0.79) |
| Cereals and cereal products | 2.42 (1.78, 3.25) | 2.67 (1.90, 3.28) | 2.31 (1.79, 3.21) | 2.57 (1.86, 3.40) | 2.22 (1.57, 2.82) | 2.75 (1.70, 3.21) | 2.82 (1.92, 3.43) | 2.89 (2.06, 3.34) |
| Refined grain products | 1.35 (0.75, 1.89) | 1.35 (0.92, 1.78) | 1.35 (0.85, 1.64) | 1.36 (0.99, 1.78) | 1.21 (0.42, 2.22) | 1.28 (0.70, 1.71) | 1.57 (0.85, 2.00) | 1.21 (0.64, 1.64) |
| Whole-grain products | 0.93 (0.50, 1.50) | 1.22 (0.61, 1.72) | 0.92 (0.42, 1.50) | 1.14 (0.49, 1.47) | 0.61 (0.39, 1.15) | 1.22 (0.57, 2.00) * | 1.36 (1.00, 2.15) | 1.50 (1.14, 1.79) |
| Milk and milk products | 1.14 (0.71, 1.57) | 1.10 (0.71, 1.61) | 1.14 (0.64, 1.50) | 1.07 (0.71, 1.50) | 1.11 (0.93, 1.61) | 0.85 (0.35, 1.21) | 1.50 (1.00, 2.21) | 1.35 (1.07, 2.00) |
| Low-fat dairy | 0.42 (0.14, 0.79) | 0.32 (0.07, 0.82) | 0.28 (0.14, 0.64) [a] | 0.21 (0.07, 0.64) | 0.46 (0.07, 0.79) | 0.25 (0.14, 0.57) | 0.89 (0.28, 1.14) [a] | 0.86 (0.49, 0.93) |
| Whole-fat dairy | 0.64 (0.43, 0.93) | 0.71 (0.43, 0.93) | 0.57 (0.28, 1.00) | 0.79 (0.43, 0.97) | 0.75 (0.57, 0.90) | 0.61 (0.21, 0.71) | 0.64 (0.28, 1.00) | 0.86 (0.28, 1.21) |
| Fats and oils | 1.07 (0.57, 1.85) | 1.21 (0.57, 2.07) | 1.43 (0.57, 1.86) | 1.18 (0.61, 1.90) | 1.00 (0.50, 1.50) | 1.28 (0.57, 1.43) | 0.75 (0.43, 1.14) | 1.50 (0.64, 2.50) |
| Oils and sauces | 0.21 (0.07, 0.64) | 0.21 (0.00, 0.56) | 0.28 (0.00, 0.79) | 0.25 (0.04, 0.57) | 0.43 (0.14, 0.65) | 0.21 (0.07, 0.78) | 0.14 (0.00, 0.28) | 0.07 (0.00, 0.21) |
| Animal fat | 0.79 (0.28, 1.50) | 0.64 (0.42, 1.28) | 0.93 (0.5, 1.500) | 0.64 (0.32, 1.29) | 0.47 (0.14, 0.90) | 0.57 (0.50, 0.86) | 0.54 (0.21, 1.07) | 0.93 (0.50, 2.50) |

Data are presented as the median and interquartile range (IQR). [a,b] $p<0.05$ For the differences between the time on the island groups (Kruskal-Wallis test);
* $p \le 0.05$, ** $p<0.01$, *** $p<0.001$ For the differences before vs. during pregnancy (Wilcoxon signed-rank test)

**Table S2. Cont.** Frequency of the intake of various food groups in women before and during pregnancy

| Intake frequencies of food groups (times/ day) | All | | Length of time living on the island | | | | | |
|---|---|---|---|---|---|---|---|---|
| | | | Native and British | | European >10y on island | | European <10y on island | |
| | Before pregnancy | During pregnancy | Before pregnancy | During pregnancy | Before pregnancy | During pregnancy | Before pregnancy | During pregnancy |
| *n* | 80 | 60 | 54 | 39 | 12 | 10 | 14 | 11 |
| Sweets, snacks, preserves | 2.19 (1.28, 3.50) | 2.35 (1.43, 3.85) | 2.27 (1.34, 4.07) | 2.57 (1.78, 4.00) | 1.46 (0.84, 3.19) | 1.31 (0.63, 2.56) | 2.20 (1.28, 3.50) | 1.78 (0.99, 3.98) |
| Fruit | 2.64 (1.57, 3.93) | 2.78 (1.92, 4.14) | 2.43 (1.35, 3.36) [a] | 2.50 (1.60, 3.54) [a*] | 2.68 (1.64, 4.40) | 3.80 (2.43, 5.27) | 4.33 (2.14, 6.16) [a] | 3.93 (2.56, 8.28) [a] |
| Vegetables | 6.13 (4.49, 7.78) | 4.57 (3.48, 6.37) ** | 5.99 (4.56, 7.91) | 4.70 (3.56, 6.15) | 4.99 (3.21, 6.62) | 3.88 (2.72, 4.37) | 6.35 (4.55, 9.23) | 5.84 (4.06, 8.45) |
| Vegetables (without legumes) | 4.93 (3.70, 6.16) | 4.56 (3.55, 6.20) | 4.65 (3.56, 6.13) | 4.56 (3.49, 5.70) | 4.40 (2.88, 5.69) | 4.27 (3.06, 4.91) | 5.79 (4.27, 8.36) | 6.93 (4.06, 9.46) |
| Legumes | 0.64 (0.28, 1.14) | 0.64 (0.28, 1.18) | 0.78 (0.42, 1.28) | 0.82 (0.35, 1.43) | 0.42 (0.28, 0.89) | 0.56 (0.42, 0.64) | 0.43 (0.21, 1.14) | 0.28 (0.14, 1.22) |
| Potatoes | 0.57 (0.35, 0.71) | 0.57 (0.42, 0.71) | 0.57 (0.35, 0.71) | 0.64 (0.43, 0.78) * | 0.39 (0.28, 0.60) | 0.50 (0.42, 0.64) | 0.68 (0.28, 1.07) | 0.50 (0.28, 0.71) * |
| Nuts and seeds | 0.14 (0.07, 0.57) | 0.14 (0.070, 0.79) | 0.14 (0.07, 0.79) | 0.25 (0.07, 0.86) | 0.14 (0.14, 0.36) | 0.11 (0.07, 0.50) | 0.14 (0.07, 0.43) | 0.14 (0.07, 0.79) |
| Non-alcoholic beverages | 3.78 (1.93, 5.50) | 3.18 (1.29, 4.58) * | 3.86 (2.14, 6.07) | 3.22 (1.32, 4.58) | 2.79 (1.61, 4.11) | 2.22 (1.21, 3.28) | 4.11 (1.28, 5.50) | 3.64 (1.28, 6.07) |
| Alcoholic beverages | 0.28 (0.07, 0.64) | 0.00 (0.00, 0.00) *** | 0.42 (0.14, 0.79) | 0.00 (0.00, 0.00) [a***] | 0.43 (0.04, 0.68) | 0.04 (0.00, 0.07) [ab*] | 0.14 (0.00, 0.35) | 0.00 (0.00, 0.00) [b*] |

Data are presented as the median and interquartile range (IQR). [a,b] $p<0.05$ For the differences between the time on the island groups (Kruskal-Wallis test);
* $p \leq 0.05$, ** p<0.01, *** p<0.001 For the differences before vs. during pregnancy (Wilcoxon signed-rank test).
